# Exploring how informed mental health app selection may impact user engagement and satisfaction

**DOI:** 10.1371/journal.pdig.0000219

**Published:** 2023-03-29

**Authors:** Marvin Kopka, Erica Camacho, Sam Kwon, John Torous

**Affiliations:** 1 Department of Psychiatry, Beth Israel Deaconess Medical Center, Boston, Massachusetts, United States of America; 2 Charité –Universitätsmedizin Berlin, corporate member of Freie Universität Berlin and Humboldt-Universität zu Berlin, Institute of Medical Informatics, Charitéplatz 1, Berlin, Germany; 3 Technische Universität Berlin, Institute of Psychology and Ergonomics (IPA), Berlin, Germany; University of Cagliari: Universita degli Studi Di Cagliari, ITALY

## Abstract

The prevalence of mental health app use by people suffering from mental health disorders is rapidly growing. The integration of mental health apps shows promise in increasing the accessibility and quality of treatment. However, a lack of continued engagement is one of the significant challenges of such implementation. In response, the M-health Index and Navigation Database (MIND)- derived from the American Psychiatric Association’s app evaluation framework- was created to support patient autonomy and enhance engagement. This study aimed to identify factors influencing engagement with mental health apps and explore how MIND may affect user engagement around selected apps. We conducted a longitudinal online survey over six weeks after participants were instructed to find mental health apps using MIND. The survey included demographic information, technology usage, access to healthcare, app selection information, System Usability Scale, the Digital Working Alliance Inventory, and the General Self-Efficacy Scale questions. Quantitative analysis was performed to analyze the data. A total of 321 surveys were completed (178 at the initial, 90 at the 2-week mark, and 53 at the 6-week mark). The most influential factors when choosing mental health apps included cost (76%), condition supported by the app (59%), and app features offered (51%), while privacy and clinical foundation to support app claims were among the least selected filters. The top ten apps selected by participants were analyzed for engagement. Rates of engagement among the top-ten apps decreased by 43% from the initial to week two and 22% from week two to week six on average. In the context of overall low engagement with mental health apps, implementation of mental health app databases like MIND can play an essential role in maintaining higher engagement and satisfaction. Together, this study offers early data on how educational approaches like MIND may help bolster mental health apps engagement.

## Author summary

Mental illnesses are common and there is a need to offer increased access to care for them. The number of mental health apps continues to grow and offers a potential solution for scalable services. Although mental health apps have grown in availability, the lack of continued engagement and satisfaction among users impedes the clinical outcome and integration. Hence, we conducted a longitudinal online survey to determine the factors affecting mental health app usage. To study this, we used the M-health Index and Navigation Database (MIND), which allows users to select through filters to find apps that meet their unique needs. Our results demonstrated that cost, condition supported by app, and app features offered were the three most important factors that users consider when selecting mental health apps. The overall engagement of the top ten apps selected also declined throughout six weeks. The results suggest the potential of app navigation towards improving engagement among mental health app users by allowing consumers to find apps that may better meet their needs.

## Introduction

For mental health apps to be effective and clinically impactful, engagement (the ongoing app use as an important step in behavior change [1]) must be maintained. A lack of engagement is multifaceted, though one core element involves matching the appropriate app to the patient’s needs. Currently, neither clinicians nor patients have training or experience recommending or selecting health apps. This paper explores pilot data around patient use of a website designed to support patients and aid them in making informed decisions regarding the selection of a mental health app.

Current data surrounding mental health app engagement is low. The landmark study by Baumel et al. in 2019 suggested that even the most popular apps lose over 80% of users within ten days [2]. Recent data on app engagement suggests similar engagement issues across a range of apps, suggesting this is a challenge not unique to any specific app [3].

Helping patients make more informed decisions around app use is a promising solution toward increasing engagement. While there are many app rating scales, the training, time, and skills to effectively use many of these have been labeled as ‘prohibitive’ [4], with one new 2022 framework requiring up to an hour to evaluate a mental health app [5]. Others have equated finding credible and useful mental health apps to finding a ‘needle in a haystack’ [6]. Curated app portals can help engender user trust and alleviate data protection concerns [7]. Yet few curated app portals exist, and those that do often struggle to update apps promptly [8].

In response, we have created a curated app portal that does not require specialized training to use and is regularly updated. The M-health Index and Navigation Database (MIND), accessible through mindapps.org, is derived from the American Psychiatric Association’s app evaluation framework [9]. This publicly available resource has been utilized by our team to conduct research [10–13] and has served as the subject of others’ research [14,15]. MIND enables users to search for apps by iteratively selecting filters that concern their goals and revealing apps that meet the specified search. For example, a user can ask to see all apps with a privacy policy, which are free and operate on Android phones as one search. Given the 105 search filters, there are numerous potential search term combinations a user can create. The features are extracted by research staff and volunteers (with changes from volunteers being evaluated and subsequently approved by research staff).

While MIND is used today by many users across the country (estimating 10,000+ website visits per month) and has been studied in academic contexts [8,16], its relation with continued app use and engagement has not been examined. While engagement remains a challenging construct to measure, the basic metric tied to use remains frequent and practical [17,18] despite flaws. In our own 2023 research, we suggest that engagement is better conceptualized as an interaction between use and alliance [19] so in this paper we present both the common metric as well as data on alliance using a validated scale [20,21]. While no studies to date have explored the association of a curated app library with app engagement, studies suggest that most users only select and engage with a small subsample of apps [22]. However, these results do not offer insights into whether app users truly engage with apps. Given the urgent need for effective interventions that sustain engagement, this study investigates 1) the factors that are associated with the use of mental health apps and 2) the association of using a database like MIND–to filter through individual preferences–with engagement and user satisfaction with a mental health app.

## Results

A total of 321 surveys were completed (178 (100%) at initial, 90 (51%) at the 2-week mark, and 53 (30%) at the 6-week mark). As seen in Table 1, the total sample was primarily White (74.5%), non-Hispanic (87.9%), and Female (77.3%). The total sample was fairly distributed in terms of income levels. However, a majority of our sample had educational attainment from a 4-year college or higher (69.8%), and 96% had health insurance. Eighty percent of the participants had been diagnosed with a mental illness, yet only 54% had used a smartphone app for their mental health in the past. Our sample was primarily comprised of Apple (52%) and Samsung (29.6%) users, see Table 1.

**Table 1:** Demographic characteristics of the sample separated into all, initial, 2-week, and 6-week surveys.

	*All Surveys*, *% (n)*	*Initial Survey*, *% (n)*	*2-Week Survey*, *% (n)*	*6-Week Survey*, *% (n)*
***Total Surveys***				
	321)	100% (n = 178) 51% (n = 90)		30% (n = 53)
***Gender Identity***				
*Female*	77.3% (n = 248)	78% (n = 139)	77.8% (n = 70)	73.6% (n = 39)
*Male*	19.6% (n = 63)	18% (n = 32)	20% (n = 18)	24.5% (n = 13)
*Non-binary*	1.9% (n = 6)	2.2% (n = 4)	1.1% (n = 1)	1.9% (n = 1)
*Transgender male*	0.9% (n = 3)	1.1% (n = 2)	1.1% (n = 1)	0% (n = 0)
*Transgender female*	0% (n = 0)	0% (n = 0)	0% (n = 0)	0% (n = 0)
*Other*	0.3% (n = 1)	0.6% (n = 1)	0% (n = 0)	0% (n = 0)
***Race***				
*White*	74.5% (n = 239)	70.8% (n = 126)	75.6% (n = 68)	84.9% (n = 45)
*Black or African American*	14% (n = 45)	16.3% (n = 29)	13.3% (n = 12)	7.5% (n = 4)
*American Indian or Alaskan Native*	1.9% (n = 6)	2.2% (n = 4)	2.2% (n = 2)	0% (n = 0)
*Native Hawaiian or other Pacific Islander*	0.3% (n = 1)	0.6% (n = 1)	0% (n = 0)	0% (n = 0)
*Asian*	5.6% (n = 18)	4.5% (n = 8)	6.7% (n = 6)	7.5% (n = 4)
*Other*	3.7% (n = 12)	5.6% (n = 10)	2.2% (n = 2)	0% (n = 0)
***Ethnicity***				
*Hispanic*	12.1% (n = 39)	12.9% (n = 23)	11.1% (n = 10)	11.3% (n = 6)
*Not Hispanic*	87.9% (n = 282)	87.1% (n = 155)	88.9% (n = 80)	88.7% (n = 47)
***Income***				
*Less than $25*,*000*	20.6% (n = 66)	23.0% (n = 41)	15.6% (n = 14)	20.8% (n = 11)
*$25*,*000-$59*,*000*	28% (n = 90)	26.4% (n = 47)	28.9% (n = 26)	32.1% (n = 17)
*$60*,*000-$84*,*000*	16.8% (n = 54)	19.1% (n = 34)	15.6% (n = 14)	11.3% (n = 6)
*$85*,*000-$99*,*000*	11.2% (n = 36)	10.1% (n = 18)	12.2% (n = 11)	13.2% (n = 7)
*$100*,*000+*	23.4% (n = 75)	21.3% (n = 38)	27.8% (n = 25)	22.6% (n = 12)
***Education level***				
*Eighth grade or less*	0.3% (n = 1)	0.6% (n = 1)	0% (n = 0)	0% (n = 0)
*Some high school*	0.6% (n = 2)	1.1% (n = 2)	0% (n = 0)	0% (n = 0)
*High school graduate/GED*	4% (n = 13)	3.9% (n = 7)	4.4% (n = 4)	3.8% (n = 2)
*Some college*	25.2% (n = 81)	28.7% (n = 51)	22.2% (n = 20)	18.9% (n = 10)
*4-year college graduate or higher*	69.8% (n = 224)	65.7% (n = 117)	73.3% (n = 66)	77.4% (n = 41)
***Phone type***				
*Apple (iPhone)*	52% (n = 167)	54.5% (n = 97)	52.2% (n = 47)	45.3% (n = 24)
*Google*	9.7% (n = 31)	7.9% (n = 14)	11.1% (n = 10)	13.2% (n = 7)
*Samsung*	29.6% (n = 95)	27.5% (n = 49)	31.1% (n = 28)	34.0% (n = 18)
*LG*	0.9% (n = 3)	0.6% (n = 1)	1.1% (n = 1)	1.9% (n = 1)
*Motorola*	5.6% (n = 18)	6.7% (n = 12)	3.3% (n = 3)	5.7% (n = 3)
*HTC*	0% (n = 0)	0% (n = 0)	0% (n = 0)	0% (n = 0)
*Other*	1.9% (n = 6)	2.2% (n = 4)	2.2% (n = 2)	0% (n = 0)
*I don’t own a smartphone*	0.3% (n = 1)	0.6% (n = 1)	0% (n = 0)	0% (n = 0)
***Wi-Fi***				
*I cannot do it on my own*	0.6% (n = 2)	1.1% (n = 2)	0% (n = 0)	0% (n = 0)
*I can do it on my own*, *but with step-by-step instructions*	0% (n = 0)	0% (n = 0)	0% (n = 0)	0% (n = 0)
*I can do it mostly on my own*, *but may have a few questions*	3.1% (n = 10)	3.9% (n = 7)	2.2% (n = 2)	1.9% (n = 1)
*I can do it on my own with ease*	16.5% (n = 53)	20.8% (n = 37)	14.4% (n = 13)	5.7% (n = 3)
*I can do it and teach someone else*	79.8%(n = 256)	74.2% (132)	83.3% (n = 75)	92.4% (n = 49)
***Download App***				
*I cannot do it on my own*	0.9% (n = 3)	1.7% (n = 3)	0% (n = 0)	0% (n = 0)
*I can do it on my own*, *but with step-by-step instructions*	0.9% (n = 3)	1.7% (n = 3)	0% (n = 0)	0% (n = 0)
*I can do it mostly on my own*, *but may have a few questions*	0.9% (n = 3)	1.1% (n = 2)	1.1% (n = 1)	0% (n = 0)
*I can do it on my own with ease*	14.0% (n = 45)	17.4% (n = 31)	12.2% (n = 11)	5.7% (n = 3)
*I can do it and teach someone else*	83.2% (n = 267)	78.1% (n = 139)	86.7% (n = 78)	94.3% (n = 50)

### Digital literacy

Comfort in one’s ability to connect to Wi-Fi was high, with initial surveys revealing that 74.2% (132/178) could do it on their own and teach someone else, and 20.8% (37/178) could do it on their own with ease but could not teach someone else (see Table 1). Over the course of the study, the participants that could do it on their own and teach others were less likely to drop out of the study and present relative risk, as seen in a percent increase from 74.2% (132/178) at initial to 92.4% (49/53) at week 6. This influence is statistically significant with an OR = 2.56 (z = 2.42, p = .016). However, the participants that could do it on their own but could not teach others were more likely to lead to attrition, as seen in a percent decrease from 20.8% (37/178) at initial to 5.7% (3/53) at week 6 –but this difference was not statistically significant (OR = 0.54, z = -1.54, p = .124).

When asked about their comfort with downloading an app from an app store, a similar trend was seen. From initial to week 6, the participants reporting they could do it on their own and teach someone else were less likely to drop out of the study, as seen in a percent increase from 78.1% (139/178) to 94.3% (50/53), while the participants that could do it on their own but could not teach others were more likely to drop out, as seen in a percent decrease from 17.4% (31/178) to 5.7% (3/53). These differences were not statistically significant (OR = 2.052, z = 1.84, p = .067 and OR = .73, z = -0.747, p = .455 respectively).

### Mental health app selection

**Fig 1:**
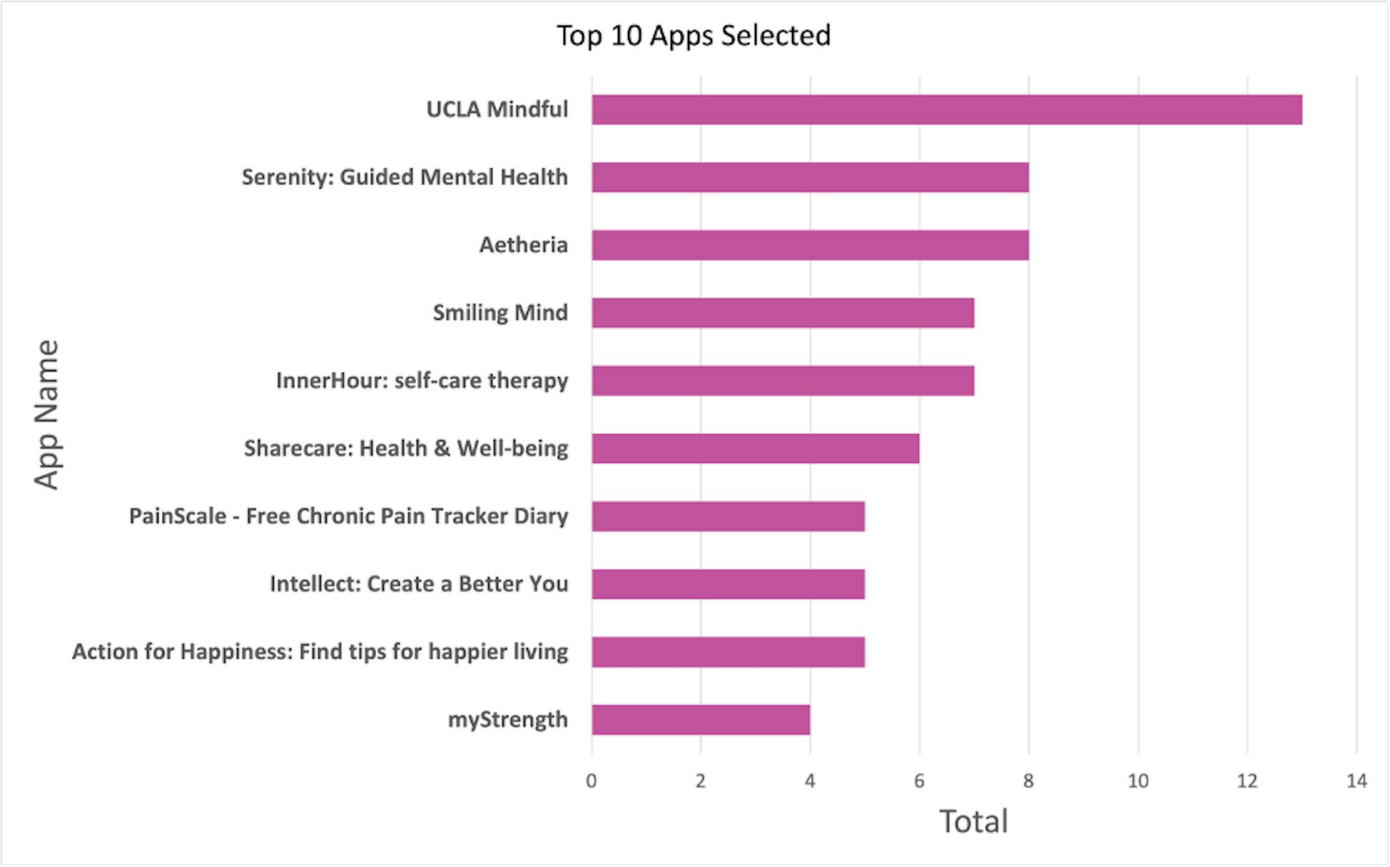
Top ten apps selected by participants.

**Fig 2:**
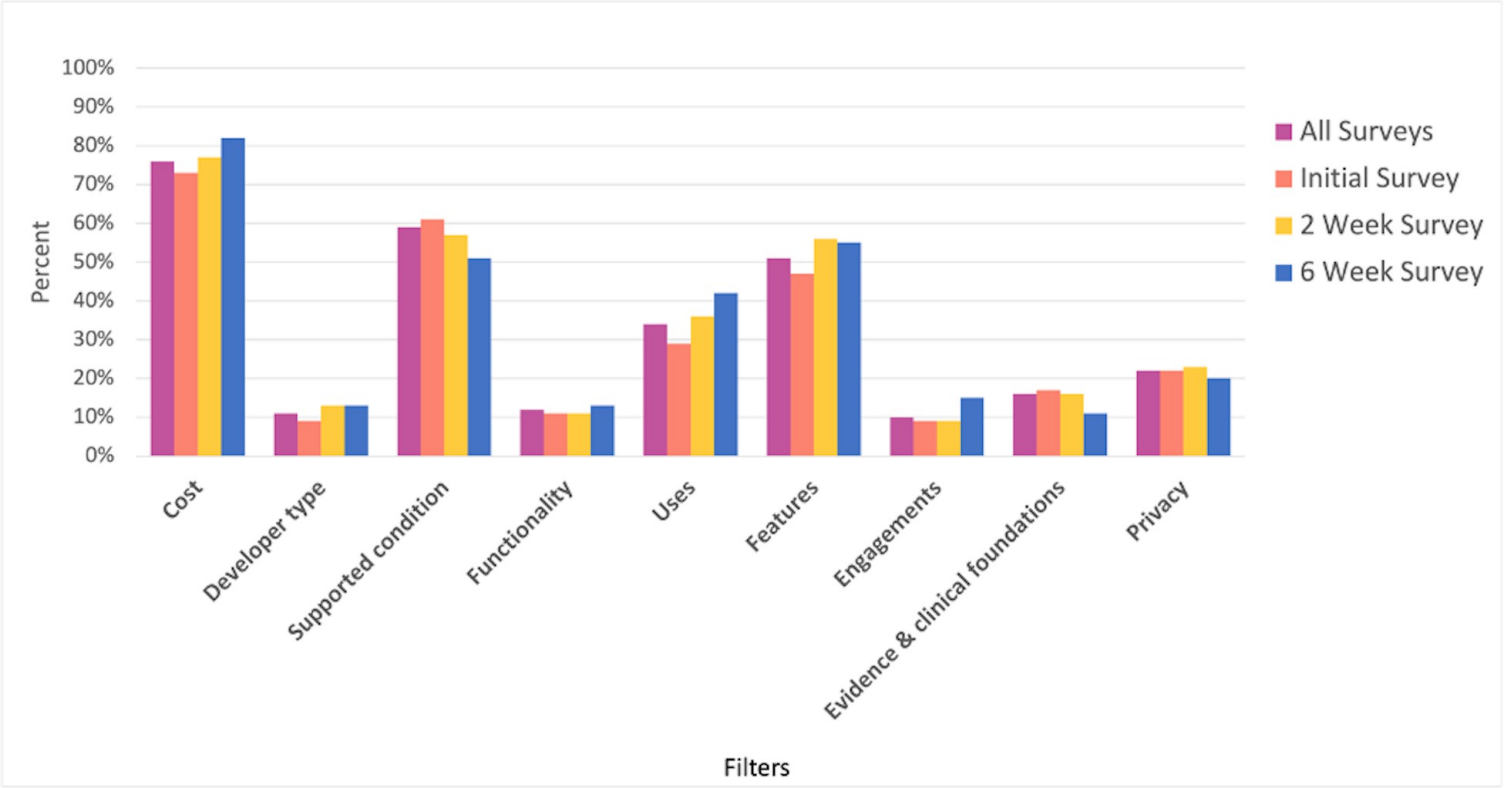
Percent of participants indicating which filters were most important in app selection.

Of the over 600 apps available for selection on the MIND database, 84 unique apps were selected by our sample to utilize for the duration of the study. The ten most selected apps (see Fig 1) comprise 38% of the app selection at initial. The top three filters informing app selections were cost (76%), condition supported by the app (59%), and app features offered (51%), as seen in Fig 2. Approximately 37% of participants reported that outside factors not included as filters in the MIND database impacted their app decision. These included reviews (55%), star ratings (43%), recommendations by clinicians or friends/family (6%), developer (4%), and number of downloads (2%) as the leading contributors.

All top ten apps were totally free (8/10) or free to download with in-app purchases (2/10). Six of the ten apps support those experiencing stress and anxiety. The primary features offered by these apps include mindfulness (9/10), goal setting (7/10), mood tracking (7/10), deep breathing (5/10), sleep tracking (5/10), and journaling (5/10). Every app was classified as a self-help app. Of the top ten apps, all offered notifications/reminders, and eight provided graphs/summaries of the data. All ten apps had a privacy policy and six of these apps allowed users to delete their data. Only two of the ten apps have published research studies to support their app capabilities; both apps have conducted feasibility and efficacy studies.

**Fig 3:**
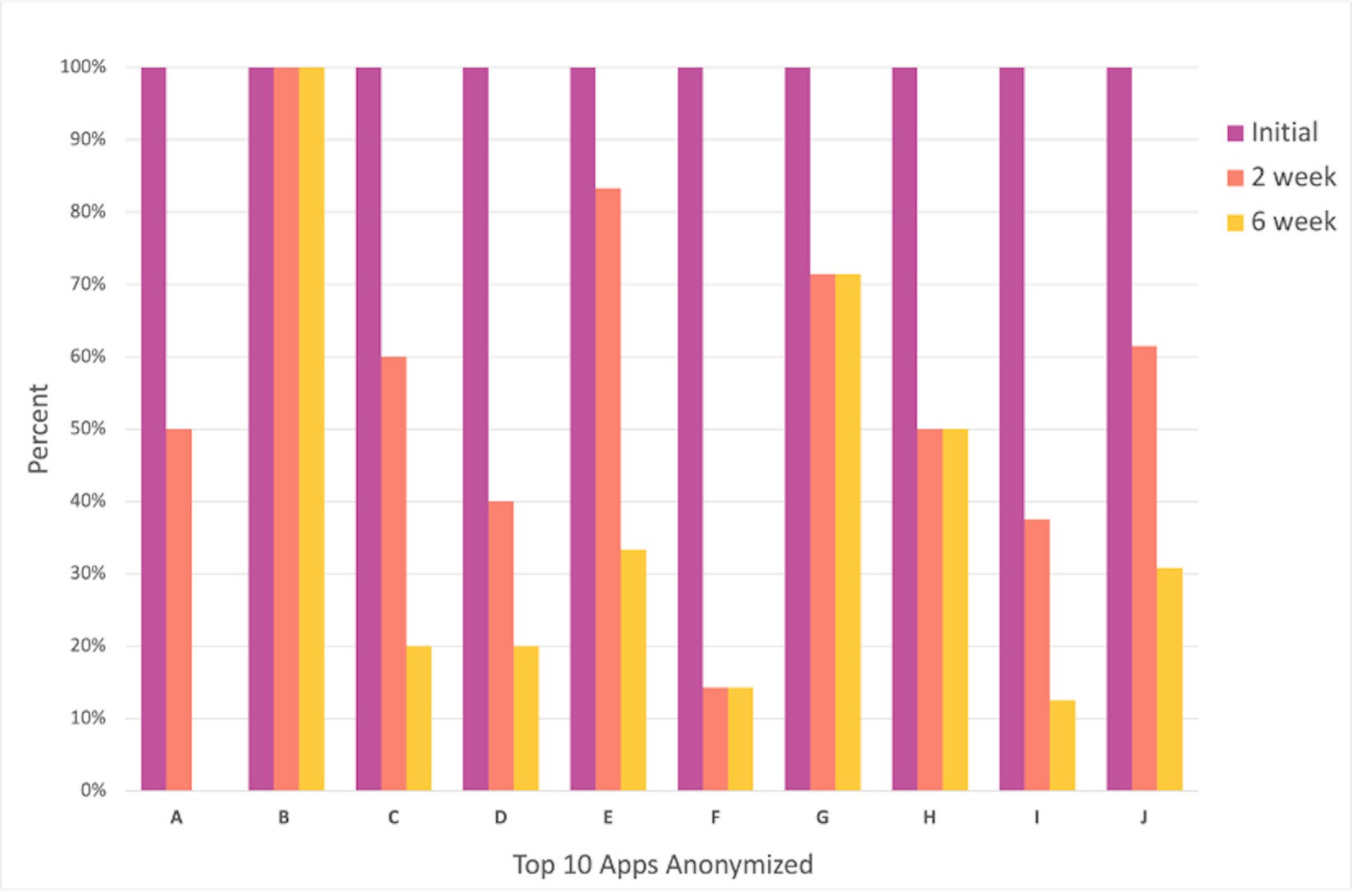
Engagement rates of participants using the top 10 selected apps.

The top ten apps were anonymized in our reporting of continued user engagement with the app they selected. As seen in Fig 3, users of one app (B) from this list reported continued use throughout all weeks of the study. One app (A) experienced a 100% loss of users throughout the study. On average, the top ten apps experienced a 43% decrease in users from initial to week two and a 22% decrease from week two to week six.

### Usability and alliance

Results across all surveys revealed high scores for SUS questions regarding interest and confidence (‘I think that I would like to use this app frequently’ and ‘I felt very confident using this app’) and low scores for questions regarding support and literacy (‘I think that I would need the support of a technical person to be able to use this app’ and ‘I needed to learn a lot of things before I could get going with this app’). Details are shown in Table 2.

**Table 2:** SUS, D-WAI, and GSE survey averages throughout study duration.

	All Surveys, Median (IQR)	Initial Survey, Median (IQR)	2 Week Survey, Median (IQR)	6 Week Survey, Median (IQR)
**Scales**				
System Usability Scale I think that I would like to use this app frequently. I think that I would need the support of a technical person to be able to use this app. I felt very confident using this app. I needed to learn a lot of things before I could get going with this app.	4 (3–5) 1 (0–2) 4 (3–5) 1 (0–2)	4 (3–5) 1 (0–2) 4 (3–5) 2 (1–3)	4 (2–6) 1 (0–2) 4 (3–5) 1 (0–2)	4 (3–5) 1 (0–2) 5 (4–6) 1 (0–2)
Digital Working Alliance Inventory Total (Mean, SD) I trust this app to guide me towards my personal goals. I believe this app tasks will help me to address my problem. This app encourages me to accomplish tasks and make progress. I agree that the tasks within this app are important for my goals. This app is easy to use and operate. This app supports me to overcome challenges.	M = 22.33 (SD = 4.9) 4 (3–5) 4 (3–5) 4 (3–5) 4 (3–5) 4 (3–5) 4 (3–5)	M = 23.00 (SD = 4.1) 4 (3–5) 4 (3–5) 4 (3–5) 4 (3–5) 4 (3–5) 4 (3–5)	M = 21.31 (SD = 5.63) 4 (3–5) 4 (3–5) 4 (2.25–5.75) 4 (3–5) 4 (3–5) 4 (3–5)	M = 21.98 (SD = 5.7) 4 (3–5) 4 (3–5) 4 (3–5) 4 (3–5) 5 (4–6) 4 (3–5)
General Self-Efficacy Scale-6 Total (Mean, SD) If someone opposes me, I can find the means and ways to get what I want. It is easy for me to stick to my aims and accomplish my goals. I am confident that I could deal efficiently with unexpected events. Thanks to my resourcefulness, I know how to handle unforeseen situations. I can remain calm when facing difficulties because I can rely on my coping abilities. I can usually handle whatever comes my way.	M = 17.01 (SD = 3.6) 3 (2–4) 3 (2–4) 3 (3–3) 3 (3–3) 3 (2–4) 3 (3–3)	M = 16.89 (SD = 6.4) 3 (2–4) 3 (2–4) 3 (3–3) 3 (3–3) 3 (2–4) 3 (3–3)	M = 17.10 (SD = 3.4) 3 (2–4) 3 (3–3) 3 (3–3) 3 (3–3) 3 (2–4) 3 (3–3)	M = 17.27 (SD = 4.2) 3 (3–3) 3 (3–3) 3 (3–3) 3 (3–3) 3 (3–3) 3 (3–3)

The D-WAI results revealed an average total score of 22.33 (SD = 4.9) across all surveys. No statistically significant changes in D-WAI total score or individual question scores were seen from initial to week 6 (t(47) = 1.60, p = .11). This remains true when controlling for digital literacy (t(45) = .67, p = .51).

The GSE showed an average total score of 17.01 (SD = 3.6) across all surveys. No significant differences were seen from study start to study end or between individual GSE question scores (t(52) = 0.96, p = .34). This remains true when controlling for digital literacy (t(49) = 1.19, p = .24).

## Discussion

This study examined 1) the drivers of mental health app selection and 2) the impact of utilizing a database of mental health apps (mindapps.org) to select apps around engagement. Findings from this study indicate that access and interest in mental health apps are high in those experiencing mental illness, with 54% of participants having used a mental health app before and 80% of participants reporting a diagnosis of a mental illness. This finding corresponds to prior literature highlighting interest in the use of mental health apps among those experiencing mental illness [23–25]. The high demand for mental health apps in our participants may be due, in part, to the high self-reported digital literacy scores and comfort with apps [26].

While rates of engagement with the selected app, as measured by engagement in this study, were low–they must be considered in light of overall low engagement with health apps. National data suggests rates of engagement may be less than 10% after two weeks [2] and numerous studies have reported similar decay curves around engagement with diverse health apps [27]. While it is not possible to directly compare engagement across studies, our results suggest that the matching process utilized in this study may potentially help increase engagement. Given the scalability of this process, larger studies with better measurement and control groups are warranted.

Our results also suggest that people are interested in a wide variety of apps. While recent studies have suggested that the majority of people use only a few mental health apps [22], our results suggest differently. Given the nature of the app stores that feature similar apps, perhaps presenting apps in no particular order and enabling people to search among all apps according to their preferences allows for the discovery of more diverse apps. A recent report on the ‘best’ mental health apps for 2022 chose apps based on evidence-based support and feature availability [28]. Of the top 13 apps listed, 11 apps were available on MIND, but only five of these were selected by participants, and none of these apps were among the top ten selected. These five apps comprise only 4% of the apps selected by the 178 participants initially. Future research exploring how education and resources can ensure people discover a wide range of apps will help ensure a diverse and healthy app ecosystem.

Existing literature on mental health app engagement suggests that users care about data privacy [29]. Results from our study revealed that the top three filters informing app selections were cost, condition supported by the app, and app features offered. Fees for the selected apps were reimbursed, but participants were asked to select an app as they would normally do. Thus, the importance of cost might even be underestimated in this study. Privacy and clinical foundation to support app claims were among the lowest selected filters for importance. Our findings indicate that accessibility in relation to cost and relevance to their specific conditions take precedence over other factors. However, this is not to say that participants do not place importance on privacy and evidence. Instead, participants reported these filters as secondary to the factors they selected. Learning more about the influence of privacy on the decision to use an app is an important target for educational interventions.

Our results can help future studies explore mechanisms in app engagement. The usability was high overall (indicating a ceiling effect), so apps that were selected appear to be highly usable. With ongoing use, the digital working alliance slightly decreased–indicating that the working alliance might be higher at initial use. Individuals with higher self-efficacy might be more likely to use the app. Future work exploring the impact of usability, alliance, and self-efficacy will help elucidate mechanisms of engagement

There were several limitations that should be acknowledged. First, our sample was predominately White, non-Hispanic females. Thus, our findings may not be representative of the general population. Additionally, the rate of attrition was high, which impacted the sample size at week 2 and week 6. However, this study was automated, and users only interacted with study staff via email, which may have reduced the length of participation. Another limitation concerns the sociodemographic characteristics of participants: we did not collect data on participants’ age and their levels of formal education were biased towards higher levels than the general US population. While eHealth users generally have higher levels of education as well, generalizability of our results to the whole US population is limited. Further, it is possible that those responding to the questionnaires were also more likely to continue using the app. Thus, participants’ engagement might have been overestimated and as noted in the introduction engagement remains a challenging construct to accurately assess. However, some participants might have also continued using the app without responding to our follow-up surveys. Communicating that the study was running for 6 weeks (without asking participants to keep using the app in that timeframe to simulate natural use) may also have had an impact on engagement. We used brief surveys in this study to encourage completion but realize full assessment scales (like the SUS) may have yielded different results. Lastly, participants were reimbursed up to $10 for apps with cost, so apps that cost more were not captured. Most apps on MIND (91%) were free, however, so this only applies to a minority of apps and we consider the influence negligible.

## Materials and methods

### Participants

This study included individuals experiencing mental illness who were recruited online through Research Match (researchmatch.org) between January 2022 to April 2022. Inclusion criteria included individuals 18 years or older, smartphone ownership, and interest in using a mental health app. The BIDMC Institutional Review Board approved a waiver of consent. Thus, participants were informed that by completing the surveys they were granting their consent.

### Outcome measures

The measured outcomes included engagement, usability, digital working alliance, and self-efficacy at each point of measurement. We measured engagement–using the response rate–by asking participants to fill out the survey only if they still used the app (after 2 and 6 weeks).

An app’s usability directly impacts the use intention and engagement with them, and users’ assessments may vary between the initial and long-term use. It was measured using a shortened version of the System Usability Scale (SUS)–a commonly used questionnaire to determine usability without addressing factors that may be irrelevant for some apps (such as offline functionality). It comprises 10 items rated on a 5-point Likert scale. The questionnaire shows acceptable validity (0.22 < r < 0.96) and reliability (α = .91) [30]. We selected four items (two for the Usable component and two for the Learnable component as proposed by Lewis & Sauro [31]) to reduce questionnaire length and attrition rates. Since we did not use the full scale, we report only individual question scores.

A therapeutic/working alliance leads to positive results, and the concept has also been applied to digital health products (called a digital working alliance). We measured it using the Digital Working Alliance Inventory (D-WAI) which quantifies the digital working alliance with smartphone interventions using a 5-point Likert scale with six questions. The questionnaire shows acceptable validity (0.26 < r < 0.75) and reliability (α = .90) [21].

Self-efficacy describes an individual’s belief to cope with difficult demands. People with higher self-efficacy might be more inclined to use an app as a result. It was measured using the short form General Self-Efficacy Scale (GSE-6) with a 4-point Likert scale and six items. The questionnaire has acceptable validity (.04 < r < .45) and reliability (α > .79) [32].

### Procedures

At the start of the six-week study, participants navigated to the MIND Database (mindapps.org) and selected filters based on a variety of categories such as cost, features, and supported conditions to discover apps of interest. Upon app selection, participants completed the initial set of surveys. Participants were informed that they will receive a survey after two and after six weeks and to respond if they still used the app. Automated emails from an approved member of the research staff were received from participants with the follow-up survey links. The surveys included questions regarding demographic information (e.g., age, gender, race, ethnicity, educational attainment, and income), technology usage (e.g., phone type, phone model, ability to connect to Wi-Fi, and ability to download an app), access to health (e.g., health insurance, whether they have a diagnosis of mental illness but the exact diagnosis), and app selection information (e.g., which filters were most important, whether outside factors informed their selection). The surveys also included the validated questionnaires described above.

No compensation was provided for participation in this study. If participants were interested in selecting an app that was $10 or less to download, they were reimbursed for that expense. This was to enable participants to select apps beyond those that are free.

### Data analysis

The data was analyzed using Excel and R Version 4.1.2. Our descriptive statistics include absolute numbers and proportions, for Likert scales means and standard deviations and for individual items the median and interquartile range. The corresponding absolute numbers and proportions were visualized in bar plots. We only included the top 10 apps, because app selection was highly heterogenous and focusing on the top 10 apps allowed us to look for broader trends.

For inferential analyses, we dichotomized the level of attrition to high (indicated by selecting “can do and teach others”) or low (other levels). We then calculated a mixed effects logistic regression with completion of follow-ups as the dependent variable and digital literacy (regarding WiFi and app downloads) as independent variables (fixed effects) and participant IDs as a random effect. For alliance and self-efficacy, we used a paired-sample t-test including only those participants who answered both initially and on week 6. As robustness checks, we included digital literacy as a covariate in a linear regression model because digital literacy was related to attrition.
